# Combined Effects of Methyldopa and Flavonoids on the Expression of Selected Factors Related to Inflammatory Processes and Vascular Diseases in Human Placenta Cells—An In Vitro Study

**DOI:** 10.3390/molecules26051259

**Published:** 2021-02-26

**Authors:** Anna Bogacz, Przemysław Ł. Mikołajczak, Marlena Wolek, Aleksandra Górska, Michał Szulc, Marcin Ożarowski, Radosław Kujawski, Bogusław Czerny, Hubert Wolski, Tomasz M. Karpiński, Agnieszka Seremak-Mrozikiewicz

**Affiliations:** 1 Department of Pharmacology and Phytochemistry, Institute of Natural Fibres and Medicinal Plants, Kolejowa 2, 62-064 Plewiska, Poland; anna.bogacz@iwnirz.pl (A.B.); przemmik@ump.edu.pl (P.Ł.M.); asm@data.pl (A.S.-M.); 2 Department of Pharmacology, Poznan University of Medical Sciences, Rokietnicka 5a, 60-806 Poznań, Poland; mszulc@ump.edu.pl (M.S.); radkuj@ump.edu.pl (R.K.); 3 Department of Stem Cells and Regenerative Medicine, Institute of Natural Fibres and Medicinal Plants, Kolejowa 2, 62-064 Plewiska, Poland; marlena.wolek@iwnirz.pl (M.W.); aleksandra.gorska@iwnirz.pl (A.G.); bczerny@wp.pl (B.C.); 4 Department of Biotechnology, Institute of Natural Fibres and Medicinal Plants, WojskaPolskiego 71b, 60-630 Poznań, Poland; 5 Department of General Pharmacology and Pharmacoeconomics, Pomeranian Medical University in Szczecin, Żołnierska 48, 70-204 Szczecin, Poland; 6 Division of Gynecology and Obstetrics, Podhale Multidisciplinary Hospital, 34-400 NowyTarg, Poland; hubertwolski@wp.pl; 7 Division of Perinatology and Women’s Diseases, Poznan University of Medical Sciences, Polna 33, 60-535 Poznań, Poland; 8 Chair and Department of Medical Microbiology, Poznań University of Medical Sciences, Wieniawskiego 3, 61-712 Poznań, Poland; tkarpin@ump.edu.pl

**Keywords:** placenta, flavonoids, inflammatory process, methyldopa

## Abstract

The aim of the study was to investigate combined effects of flavonoids (apigenin, baicalein, chrysin, quercetin, and scutellarin) and methyldopa on the expression of selected proinflammatory and vascular factors in vitro for prediction of their action in pregnancy-induced hypertension. The research was conducted on a trophoblast-derived human choriocarcinoma cell line and a primary human umbilical vein endothelial cell line. Cytotoxicity of compounds in selected concentrations (20, 40, and 100 µmol) was measured using the MTT test and the concentration of 40 µmol was selected for further analysis. Subsequently, their effects with methyldopa on the expression of selected markers responsible for inflammation (TNF-α; IL-1β; IL-6) and vascular effects (hypoxia-inducible factor 1α—HIF-1α; placental growth factor—PIGF; transforming growth factor β—TGF-β; vascular endothelial growth factor—VEGF) at the mRNA and protein levels were assessed. It was found that every combined administration of a flavonoid and methyldopa in these cells induced a down-regulating effect on all tested factors, except PIGF, especially at the mRNA expression level. As hypertension generally raises TNF-α, IL-1β, IL-6, HIF-1α, TGF-β, and VEGF mRNA expression and/or protein levels, the results obtained in the studied model may provide a positive prognostic factor for such activity in vivo.

## 1. Introduction

Preeclampsia, a hypertensive disorder of pregnancy, is one of the leading causes of maternal and fetal morbidity and mortality. The disease is a cause of serious consequences, including impairments of multiple organ systems (central nervous, hepatic, pulmonary, renal, and hematologic systems) and the leading cause of maternal mortality in both developed and developing countries. Moreover, fetal complications include placental abruption, intrauterine growth restriction, premature delivery, and intrauterine fetal death [1,2,3].

The etiology of preeclampsia remains not fully understood [4,5]. Preeclampsia likely begins at implantation with superficial invasion of placenta vessels leading to excessive release of physiological antiangiogenic factors [6]. According to this theory, abnormal spiral artery remodeling in early pregnancy causes placental hypoxia. The ischemic placenta releases into the maternal circulation large amounts of soluble factors, such as reactive oxygen species, pro-inflammatory cytokines, and anti-angiogenic factors, which lead to the clinical manifestations of the disease [7,8].

It is known that in normal pregnancy, systemic inflammation, oxidative stress, and alterations in levels of angiogenic factors and vascular reactivity are observed, but this process is exacerbated in preeclampsia with associated breakdown of compensatory mechanisms, eventually leading to placental and vascular dysfunction. One hypothesis concerns placental endothelial cell dysfunction [4,9]. Changes in endometrial levels of many angiogenic growth factors have been observed in pregnancy-induced hypertension (PIH), including vascular endothelial growth factor A (VEGF-A) and placental growth factor (PIGF) [10,11].

Preeclampsia is also characterized by excessive and progressive activation of the immune system along with increases in proinflammatory cytokines and antiangiogenic factors in the fetoplacental unit andan increase in vascular endothelium in pregnant women. A single, major underlying mechanism of preeclampsia is yet to be identified. Inflammation is an active process regulated by various mediators that control key cellular events to restore tissue homeostasis. Impaired resolution of inflammation probably plays a vital role in the development of chronic inflammatory diseases, and preeclampsia is believed to be one of them [12]. It is suggested that preeclamptic women display an exaggerated inflammatory response in the course of pregnancy due to unbalanced regulation of innate and adaptive immune responses [13].

Much attention is paid to the use of antihypertensive drugs from different pharmacological groups in pregnancy [14]. Drug treatment options in preeclampsia are limited because some antihypertensive drugs, such as angiotensin converting enzyme (ACE) inhibitors and angiotensin II receptor (AT1) antagonists (sartans), have shown teratogenic effects on the fetus [15]. Methyldopa belongs to the group of drugs acting on the central nervous system to evoke depression in the cardiovascular system [16]. Numerous reports have confirmed the great usefulness of this drug for reducing blood pressure in pregnant women, both in preeclamptic women and in women with chronic hypertension [16]. The reduction of blood pressure in pregnant women with hypertension using methyldopa can have a significant positive impact on the uteroplacental circulation, which improves the provision of oxygen and nutrients to the developing fetus [17,18,19]. This drug remains a mainstay of preeclampsia treatment mostly due to reported uteroplacental perfusion stability and fetal hemodynamics [20,21]. However, treatment of preeclampsia remains sometimes challenging, since methyldopa has some adverse side effects, such as hepatotoxicity [22], and may be hard to tolerate due to causing dizziness, depression, or headache [23].

One possibility of obtaining a stronger antihypertensive effect with possible reduction of side effects is combined therapy [24]. This kind of therapy shows a greater chance of obtaining a hypotensive response in a pathogenically complex disease such as preeclampsia, due to different mechanisms of drug action, greater potency of the hypotensive effect due to synergistic effects, and the possibility of using drugs in doses that give the lowest possible likelihood of adverse effects. Therefore, the search for new drugs for use in combined therapy is necessary. One source of new drugs is herbal plants, which include known plant materials with proven antihypertensive effects [25]. A growing number of studies indicate the cardioprotective effects of flavonoids—natural polyphenolic compounds commonly found in fruits, vegetables, and beverages. It is well known that such flavonoids as quercetin, apigenin, and chrysin are found in many medicinal plants, for example, buckwheat (*Fagopyrum esculentum* Moench), hawthorn (*Crataegus* spp.), and passionfruit (*Passiflora* spp.) [26]. Baicalein and scutellarin are characteristic of the genus *Scutlaria* (for example, in Baikal skullcap (*Scutellaria baicalensis* Georgi), although they were also found in other plants: baicalein in Indian trumpetflower (*Oroxylum indicum*) [27] and thyme (*Thymus vulgaris* L.) [28]; scutellarin in Erigeron breviscapus [29]. It is known that quercetin, a flavonoid well known for its antihypertensive action, may be considered a prototype for a safe antihypertensive drug [2]. Moreover, other flavonoids such as quercetin [30], apigenin [31], chrysin [32], baicalein [33,34], and scutellarin [35] exhibit vasoprotective properties, and many other activities, such as anti-oxidation via several pathways, anti-inflammation, anti-ischemia, cardioprotection, and anti-hypertension [36]. These flavonoids have not demonstrated teratogenic or abortive effects, so they are generally recognized as safe [37,38].

Although the flavonoids possess antihypertensive activities via various mechanisms of action [2,36], so far, data on their effects in pregnancy-induced hypertension are generally unavailable. To address this problem, in this study we decided to investigate the effects of the combined administration of each of the tested flavonoids with methyldopa in vitro—using cells that were derived from human trophoblasts (JEG-3 cells) and human umbilical vein endothelial cells (HUVEC)—on the expression of selected markers responsible for inflammation (tumor necrosis factor α—TNF-α; interleukin 1—IL-1β; interleukin 6—IL-6) and vascular effects (hypoxia-inducible factor 1α—HIF-1α; placental growth factor—PIGF; transforming growth factor β—TGF-β;vascular endothelial growth factor—VEGF) at the mRNA and protein levels.

## 2. Results

### 2.1. Cell Viability

**Figure 1:**
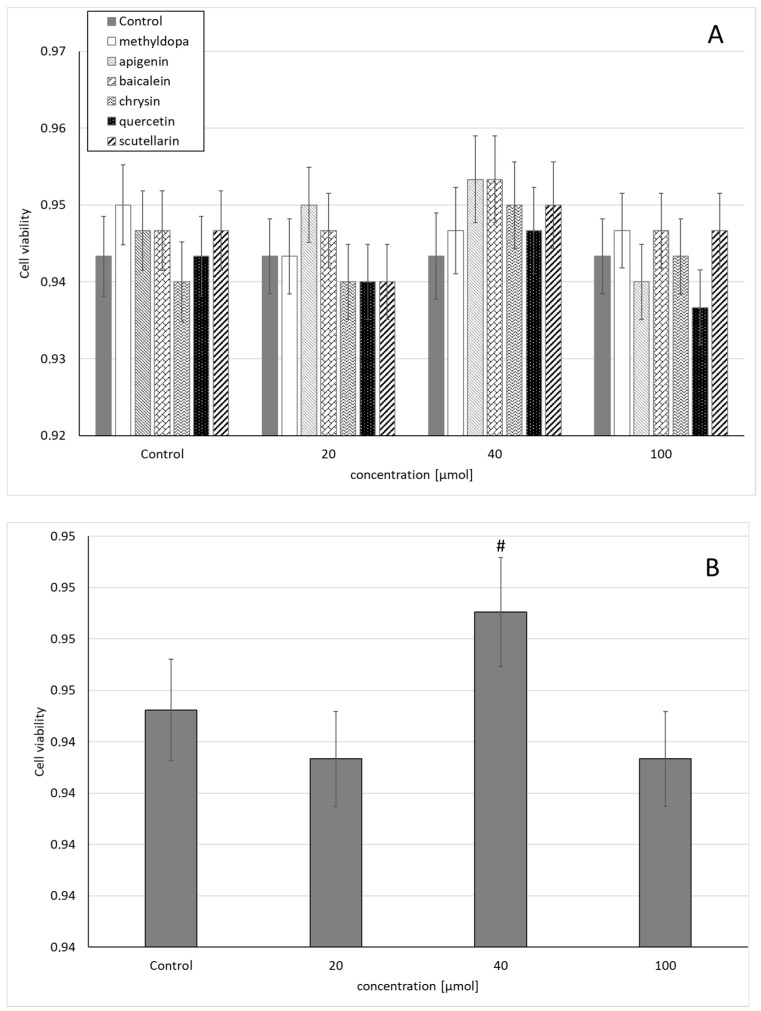
Effects of methyldopa and individual flavonoids on cell viability using the MTT test in JEG-3 cells. (**A**) Dose-dependent effectsof individual groups. (**B**) Dose-dependent effects of groups of all compounds considered for given concentrations; mean ± SD, #—vs. control, *p* < 0.05.

Statistical analysis of the survival of JEG-3 cells exposed to separate solutions containing each of the individual flavonoids, i.e., apigenin, baicalein, chrysin, quercetin, and scutellarin, and methyldopa alone at increasing concentrations (20, 40, and 100 μmol) relative to the vehicle receiving group (control = DMSO) showed no statistically significant differences in variability of survival (MTT test) between concentrations (main effect) and compounds (grouping effect) (analysis of variance—ANOVA, interaction: F (18,42) = 0.407, *p* = 0.9789) (Figure 1A). However, analysis of the variability of the main effect, when groups of all compounds were considered for a given concentration (ANOVA, main effect: F(3,42) = 2.121, *p* = 0.1118), showed that the concentration of 40 μmol showed a small, but statistically significant increase in survival compared to the control group (*p* < 0.05) (Figure 1B).

**Figure 2:**
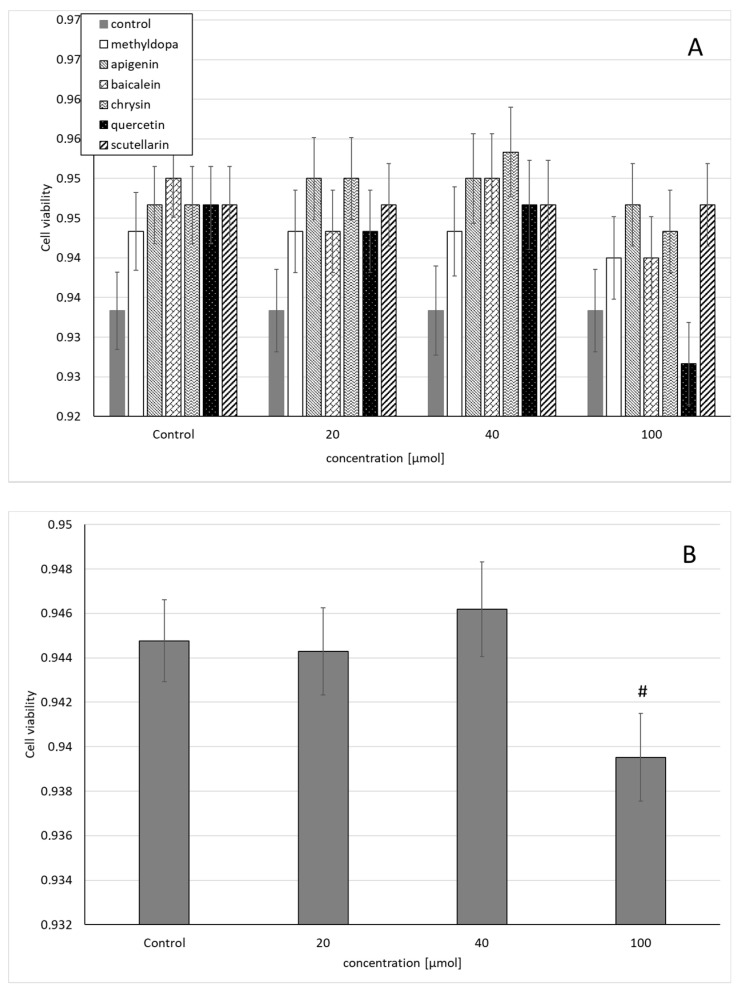
Effects of methyldopa and individual flavonoids on cell viability using the MTT test in human umbilical vein endothelial cells (HUVEC) cells. (**A**) Dose-dependent effects of individual groups. (**B**) Dose-dependent effects of groups of all compounds considered for given concentrations; mean ± SD, #—vs. control, *p* < 0.05.

Similarly, the statistical analysis of the survival of HUVEC exposed to separate solutions containing each of the individual flavonoids, i.e., apigenin, baicalein, chrysin, quercetin, and scutellarin, and a solution of methyldopa alone at increasing concentrations (20, 40, and 100 μmol) relative to the vehicle-receiving group (control = DMSO) showed no statistically significant differences in variability in survival (MTT test) between concentrations (main effect) and compounds (grouping effect) (ANOVA, interaction: F(18,42) = 0.537, *p* = 0.9228) (Figure 2A). However, analysis of the overall variability when groups of all compounds were considered for a given concentration (ANOVA, main effect: F(3,42) = 2.272, *p* = 0.0941) showed that the concentration of 40 μmolallowed a small but statistically insignificant increase in survival compared to the control (*p* < 0.05), but in the group receiving 100 μmol, reduced survival was observed, statistically significantly in relation to the control group (Figure 2B).

Therefore, for all analyzed compounds a concentration of 40 μmol was selected for further analysis. After choosing the concentration of 40 μmol as optimal, the effects of their separate and combined application with methyldopa (40 μmol) on the expression of TNF-α, TGF-β, VEGF, PIGF, HIF-1 α, IL-1β, and IL-6 both at mRNA and protein levels in JEG-3 and HUVEC cells were measured. A group of cells that received methyldopa served as a positive control.

### 2.2. TNF-Alpha

#### 2.2.1. Studies Conducted on Human Trophoblasts of JEG-3 Cells

In the studies on the influences of the tested compounds on the expression of TNF-α mRNA and protein, statistically significant overall variabilities were observed (ANOVA: F(11,12) = 17.3, *p* < 0.0001; F(11,12) = 6.98, *p* < 0.01, respectively). It was found that methyldopa and all flavonoids reduced expression similarly compared with the control group (by about 15–17%); only chrysin was more potent (by 25% and 35% on mRNA and protein levels, respectively) (Table 1 and Table 2). On the other hand, the administrations of flavonoids together with methyldopa showed stronger decreases in expression compared to the control values, and such combinations had stronger inhibitory effects when compared with methyldopa itself, which was noted for all compounds, although the strongest effects were found for the combinations of methyldopa + chrysin, methyldopa + scutellarin (a decrease by about 50% in mRNA) (Table 1),and methyldopa + quercetin (decrease by 35% on protein level)(Table 2).

#### 2.2.2. Studies Carried Out on Human Umbilical Vein Endothelial Cells (HUVEC)

In studies on the influence of the tested compounds on TNF-α mRNA and protein expression, statistically significant overall variabilities were found (ANOVA: F(11,12) = 2.30, *p* < 0.05; F (11,12) = 23.9, *p* < 0.0001, respectively). It was found that methyldopa and all flavonoids increased the mRNA expression similarly (by about 40%); only apigenin was more potent (by 94%) (Table 3). On the protein level, lowered expression compared to the control group was observed, although a significant effect was found only for methyldopa, apigenin, baicalein, and quercetin (by about 13%)—for the latter the strongest effect was noted (decrease by 24%) (Table 4). The administrations of flavonoids together with methyldopa showed inhibitory effects on mRNA and protein expression compared to the values for methyldopa and led to values similar to the control group, butthe strongest effect was found for the combination of methyldopa + scutellarin on the mRNA level (a decrease of about 24% compared to methyldopa; Table 3) and formethyldopa + baicalein on the protein level (by 32% compared with methyldopa values) (Table 4).

### 2.3. IL-1Beta

#### 2.3.1. Studies Conducted on Human Trophoblasts of JEG-3 Cells

In the studies on the influence of the tested compounds on the expression of IL-1βat mRNA and protein levels, the existence of statistically significant overall variabilitieswas found (ANOVA: F(11,12) = 25.8, *p* < 0.0001; F(11,12) = 4.63, *p* < 0.01, respectively). Detailed analysis on the mRNA level showed that methyldopa and all flavonoids had lower expression, although significant effects were noted only for methyldopa (13%), apigenin (15%), and chrysin (18%) (Table 1), whereas on the protein level there was slightly increased expression compared to the control group, although it was not significant (Table 2). The administration of every flavonoid and methyldopa combination showed an inhibitory effect on mRNA expression compared to the value for methyldopa, and the values obtained were from less than 32% (quercetin) to 44% (chrysin) (Table 1), but on the protein level, opposite effects were noticed. The strongest increase of IL1βat the protein level was for the combination of methyldopa + baicalein in relation to methyldopa alone (by 90%) (Table 2).

#### 2.3.2. Studies Carried Out on Human Umbilical Vein Endothelial Cells (HUVEC)

In studies on the influences of the tested compounds on the expression of IL-1βat mRNA and protein levels, statistically significant overall variabilities were observed (ANOVA: F(11,12) = 8.64, *p* < 0.0001; F(11,12) = 3.43, *p* < 0.05, respectively). It was found that methyldopa and all flavonoids increased expression, and the strongest effect was found for apigenin (by 73%) (Table 3). At the protein level, methyldopa lowered the level of IL-1β, while flavonoids increased the values, but the differences in most of the comparisons did not reach statistical significance; only scutellarin significantly increased the amount of this interleukin (by 33%) (Table 4). Similarly, the administration of every flavonoid and methyldopa combination also showed an increase in mRNA expression, and the strongest effect compared to the value for methyldopa was noted for the combination of methyldopa + chrysin (20% increase vs. methyldopa) (Table 3). At the protein level, the administration of flavonoids together with methyldopa showed mostly a slight decrease in the value of IL-1β, but none of the differences in the methyldopa ratio were statistically significant (Table 4).

### 2.4. IL-6

#### 2.4.1. Studies Conducted on Human Trophoblasts of JEG-3 Cells

In the studies on the influences of the tested compounds on the expression of IL-6 at mRNA and protein levels, the existence of statistically a significant general variabilityfor mRNA was observed (ANOVA: F (11,12) = 35.6, *p* < 0.0001), whereas at the protein level no statistically significant general variability was observed (ANOVA: F(11,12) = 1.99, *p* > 0.01). It was found that methyldopa and all flavonoids increased mRNA expression, but a significant effect was found only for apigenin (by 20%), baicalein (by 18%), and scutellarin (by 30%) (Table 1). Moreover, the administration every combination of a flavonoid and methyldopa showed different inhibitory effects for all flavonoids compared to the value for methyldopa, which all occurred at a similar level of 42–45%.At the protein level, the effects of the administration of the compounds individually and their combined administration with methyldopa did not reach statistical significance compared to the respective control groups (Table 2).

#### 2.4.2. Studies Carried Out on Human Umbilical Vein Endothelial Cells (HUVEC)

In the studies on the influences of the tested compounds on the expression of IL-6 at the mRNA and protein levels, the existence of statistically significant general variabilitieswas observed (ANOVA: F(11,12) = 45.7, *p* < 0.0001; F(11,12) = 22.6, *p* < 0.0001, respectively). It was found that methyldopa and all flavonoids similarly increased the expression, and the strongest significant effect was found for methyldopa (by 45%), followed by apigenin and quercetin (by 23%) (Table 3). At the protein level, the flavonoids induced a significant increase ranging from 12% (apigenin) to 42% (scutellarin) (Table 4). However, the administrations of flavonoids together with methyldopa showed significantly different inhibitory effectson mRNA compared to the value for methyldopa alone; the former effects ranged from 41% (methyldopa + apigenin) to 45% (methyldopa + quercetin) (Table 3). An inhibitory effect was also found at the protein level, which ranged from 26% (methyldopa + apigenin) to 35% (methyldopa + quercetin or methyldopa + baicalein) (Table 4).

### 2.5. TGF-β

#### 2.5.1. Studies Conducted on Human Trophoblasts of JEG-3 Cells

In studies on the influences of the tested compounds on the expression of TGF-βat the mRNA and protein levels, the existence of statistically significant general variabilities was observed (ANOVA: F(11,12) = 15.9, *p* < 0.0001; F(11,12) = 92.6, *p* < 0.0001). It was found that methyldopa and all flavonoids actedsimilarly regarding mRNA, and did not significantly affect mRNA expression in relation to the control group (Table 1). At the protein level, it was found that methyldopa, apigenin, baicalein, and quercetin significantly decreased TGF-β levels, whereas scutellarin and chrysin increased the levels (Table 2). On the other hand, the administration of every flavonoid significantly lowered mRNAexpression in relation to the methyldopa group, showing effects of 41% (methyldopa + apigenin and methyldopa + quercetin) to 57% (methyldopa + chrysin) (Table 1). At the protein level, similar effects were observed, and the strongest inhibitory activity was noted for methyldopa + quercetin compared to methyldopa alone (by 16%) (Table 2).

#### 2.5.2. Studies Carried Out on Human Umbilical Vein Endothelial Cells (HUVEC)

In studies on the influences of the tested compounds on the expression of TGF-β mRNA and the protein level, statistically significant overall variabilities were observed (ANOVA: F(11,12) = 4.63, *p* < 0.001; F(11,12) = 335.78, *p* < 0.0001). It was found that methyldopa and all flavonoids acted similarly in their degree of significantly increasing mRNA expression in relation to the control group: from 25% (quercetin) to 52% (scutellarin) (Table 3). However, methyldopa (by 22%) and quercetin (by 36%) significantly decreased the level of TGF-β, whereas baicalein increased it (by 13%), and the remaining compounds, chrysin and scutellarin, did not differ significantly from the control values (Table 4). On the other hand, every combined administration of flavonoids significantly lowered the expression in relation to the methyldopa group, showing an inhibitory effect, which was most apparent for the combination: methyldopa + quercetin and methyldopa + chrysin groups (by 25%) (Table 3). Similar effects were found at the protein level, andthe strongest inhibitory effects were noted for methyldopa + quercetin and methyldopa + baicalein compared to methyldopa alone (by 32%) (Table 4).

### 2.6. VEGF

#### 2.6.1. Studies Conducted on Human Trophoblasts of JEG-3 Cells

In studies on the influences of the tested compounds on the expression of VEGF mRNA and the protein level, statistically significant overall variabilities were demonstrated (ANOVA: F(11,12) = 18.5, *p* < 0.0001; F(11,12) = 18.5, *p* < 0.0001, respectively). It was observed that methyldopa and all flavonoids acted similarly in their degree of increasing mRNA expression compared to the control group, but only the effects of methyldopa (by 37%), apigenin (by 28%), and scutellarin (by 33%) were significant (Table 1), whereas at the protein level, the results were not homogeneous, since methyldopa (by 65%), baicalein (by 21%), quercetin (by 25%), and scutellarin (by 12%) significantly decreased the level of VEGF, but apigenin and chrysin increased it (by 17% and 11%, respectively) (Table 2). On the other hand, every combination of flavonoids significantly lowered the mRNA expression compared to the methyldopa group, showing an inhibitory effect, which ranged in inhibitory action from 46% (methyldopa + quercetin) to 66% (methyldopa + chrysin) (Table 1). Similar effects were shown at the protein level, since the combined administration of methyldopa + quercetin showed an inhibitory effect in relation to the action of methyldopa alone (by 22%); for the remaining flavonoids, the effects of combinations with methyldopa were weaker in relation to the action of the flavonoids alone, but they exceeded the effect caused by methyldopa (Table 2).

#### 2.6.2. Studies Carried Out on Human Umbilical Vein Endothelial Cells (HUVEC)

In studies on the influences of the tested compounds on the expression of VEGF at the mRNA and protein levels, statistically significant overall variabilities were observed (ANOVA: F(11,12) = 2.41, *p* < 0.05; F(11,12) = 267.5, *p* < 0.0001). It was found that methyldopa and all flavonoids acted similarly in their degree of significantly increasing mRNA expression in relation to the control group: 25% (chrysin), 28% (methyldopa), and 51% (scutellarin) (Table 3). At the protein level, only apigenin significantly increased the level of VEGF (by 24%), while the values for all the other compounds were not interchangeable with the values for the control group (Table 4). On the other hand, every combination of flavonoids lowered the mRNA expression in relation to the methyldopa group, showing an inhibitory effect, but the differences were not statistically significant (Table 3). Similarly, at the protein level, the administrations of methyldopa and individual flavonoids decreased the level of VEGF in relation to the effect of methyldopa, and the obtained values were lower by 16% (methyldopa + chrysin) up to 71% (methyldopa + quercetin) (Table 4).

### 2.7. PIGF

#### 2.7.1. Studies Conducted on Human Trophoblasts of JEG-3 Cells

In studies on the influences of the tested compounds on the expression of PIGF mRNA and protein, statistically significant overall variabilities were observed (ANOVA: F(11,12) = 7.74, *p* < 0.0001; F(11,12) = 5.63, *p* < 0.01). It was noted that methyldopa and all flavonoids acted similarly in their degree of increasing mRNA expression, but only for methyldopa and apigenin was the difference statistically significant in relation to the control group (Table 1). At the protein level, only methyldopa significantly decreased PIGF levels (by 24%), while the values for all other compounds were not interchangeable with the values for the control group (Table 2). On the other hand, every combination of flavonoids lowered the mRNA expression in relation to the methyldopa group, showing an inhibitory effect, but the differences were not statistically significant (Table 1). On the contrary, the administrations of methyldopa and individualflavonoids increased the protein level of PIGF in relation to the effect of methyldopa; the values were the highest for methyldopa + baicalein (by 13%) (Table 2).

#### 2.7.2. Studies Carried Out on Human Umbilical Vein Endothelial Cells (HUVEC)

In the studies on the influences of the tested compounds on the expression of PIGF at the mRNA and protein levels, statistically significant overall variabilities were observed (ANOVA: F (11,12) = 3.70, *p* < 0.01 and F(11,12) = 913.1, *p* < 0.0001). It was noted that methyldopa and all flavonoids acted similarly in their degree of increasing mRNA expression, and the strongest effectswere noted for methyldopa (by 62%) and scutellarin (by 48%) compared to the control group (Table 3). Similarly, at the protein level all flavonoids significantly increased the level of PIGF, while for methyldopa no such effect was observed compared to the control group, and the highest value was measured for baicalein (by 5%). (Table 4). Every combination of flavonoids significantly lowered the mRNA expression and protein level in relation to the methyldopa group, showing an inhibitory effect though, and the strongest inhibitory effect was found for the combination methyldopa + quercetin (mRNA and protein levelsby 30% and 17%, respectively) (Table 3 and Table 4).

### 2.8. HIF-1

#### 2.8.1. Studies Conducted on Human Trophoblasts of JEG-3 Cells

In studies on the influences of the tested compounds on HIF-1 at the mRNA and protein levels, they showed statistically significant general variabilities (ANOVA: F(11,12) = 20.4, *p* < 0.0001; F (11.12) = 14.4, *p* < 0.0001). It was noted that methyldopa and all flavonoids acted similarly in their degree of increasing mRNA expression, but only for methyldopa was the difference statistically significant in relation to the control group (Table 1). A similar effect was observed at the protein level (Table 2). On the other hand, the administration of every flavonoid and methyldopa combination lowered the mRNA expression in relation to the methyldopa-only group. The strongest inhibitory effectswere observed for the combinations of methyldopa + baicalein (by 35%) and methyldopa + chrysin (by 36%) (Table 1). Similar effects were observed at the protein level, and the strongest inhibitory effects were observed for the combinations of methyldopa + quercetin and methyldopa + chrysin (by 77% and 72%, respectively), leading to values similar to those obtained in the control group (Table 2).

#### 2.8.2. Studies Carried Out on Human Umbilical Vein Endothelial Cells (HUVEC)

The studies on the influences of the tested compounds on the expression of HIF-1 mRNA and protein levelsshowed statistically significant general variabilities (ANOVA: F(11,12) = 41.9, *p* < 0.0001; F(11,12) = 7.96, *p* < 0.001). It was found that methyldopa and all flavonoids act similarly in their degree of increasing mRNA expression, and the strongest effect was noted for apigenin (by 70%), followed by scutellarin (by 48%), when compared to the control group (Table 3). Similar effects at the protein level were noted, and statistically significant differences in the values for methyldopa (by 47%), baicalein (by 47%), scutellarin (by 41%), and chrysin (most potent—by 76%) were obtained when compared with control group (Table 4). On the other hand, every combined administration of a flavonoid with methyldopa lowered the mRNA expression in relation to the methyldopa group, showing an inhibitory effect, and all obtained values differed significantly from the values for methyldopa (*p* < 0.01), leading to values similar to those obtained in the control group (Table 3).At the protein level, results were less homogenous, since the combined administrations of methyldopa and most individual flavonoidsinsignificantly decreased the values of HIF-1 in comparison to the effect of methyldopa. Only in the case of combined administration of methyldopa + scutellarin was a significant increase in the level of HIF-1 (by 20%) found when compared with methyldopa (Table 4).

## 3. Discussion

Although it is known that in vitro tests will not exactly match the processes occurring in the entire tissue or body, the use of models based on the cells of the obtained target tissues allows one to approximate the phenomena occurring in vivo [39]. Therefore, the studies were carried out using human JEG-3 cell trophoblasts and human umbilical vein endothelial cells (HUVEC). JEG-3 cells were used as a model for the investigation of changes in placenta trophoblasts according to other studies [39,40,41], whereas HUVEC were used as a model for the investigation of changes in endothelial cells [42].

In the first stage, the influences of methyldopa and the tested flavonoids on the cytotoxicity of compounds were measured using an evaluation of overall survival rate with the MTT technique [43]. It was found that all compounds act similarly in the range of 20–100 μmol, and in practice no significant differences in overall survival rate were observed between the concentrations of all compounds, although for both cell systems used (JEG-3 and HUVEC) when analyzed together (main effect versus dose), the lowest cytotoxicity was noted at 40 μmol. Therefore, for further research, this concentration was used both for substances administered separately and in combination with methyldopa. Detailed understanding of the pathophysiology of the inflammatory process in preeclampsia is still a subject of research [44,45]. It is known that TNF-α is a central regulator of inflammation, and this cytokine plays a crucial role in causing inflammation predominantly by means of T lymphocytes. It is also associated with inflammatory mechanisms related to implantation, placentation, and pregnancy outcome, since overproduction of TNF-α may lead to such events as recurrent pregnancy loss, early and severe pre-eclampsia, and recurrent implantation failure syndrome [46]. In our study it was found that all the compounds tested, i.e., methyldopa and all flavonoids, reduced the expression of TNF-α at both the mRNA and protein levels to similar extents in JEG-3 cells. In these cells the combined administration any flavonoid together with methyldopa showed stronger decreases in TNF-α mRNA expression and protein presence, and the strongest effect was found for methyldopa + scutellarin. On the other hand, in HUVEC cells all the compounds tested, i.e., methyldopa and all flavonoids, increased mRNA TNF-α expression to a similar extent, whereas their combined treatment with methyldopa lowered the expression and led to values similar to the control. At the protein level, less visible effects were noted, although combined administration also produced control-like effects. It is known that TNF-α inhibits trophoblast and endothelial cellular interactions and simultaneously decreases endothelial nitric oxide synthase (eNOS) expression, and methyldopa reversed TNF α-induced inflammation and increased eNOS expression in vitro [47]; our effects ofmethyldopa may be consistent with these observations.

It is known that IL-1 is a possible mediator of maternal endothelial dysfunction in preeclampsia [48,49], and aberrant IL-1β levels were shown to be associated with a variety of gestational diseases, such as preeclampsia, preterm labor, and spontaneous abortion [50]. It is known that IL-1 is elevated in maternal blood from women with pre-eclampsia [51]. We observed that all the compounds tested, i.e., methyldopa and all flavonoids, lowered the mRNA IL-1β expression, and every combined flavonoid and methyldopa treatment showed changes in the same direction in JEG-3 cells. However, at the protein level the effects were slight and not significant, but the administrations of flavonoids together with methyldopa showed stronger increases in expression; in particular, the combination of methyldopa + baicalein produced a very strong effect. In HUVEC cells it was found that all the compounds tested, i.e., methyldopa and all flavonoids, increased mRNA expression, and flavonoids’combined treatment with methyldopa strengthened this effect. In contrast, flavonoids together with methyldopa mostly showed mostly slight decreases in the value of IL-1β at the protein level, but none of the differences in the methyldopa ratio were statistically significant. These somewhat surprising and opposite results obtained depending on the type of cells and the measurement of mRNA or protein levels are difficult to explain; however, in studies on women different results were obtained. For example, onset of labor results in elevations in amniotic fluid levels of IL-1β that are similar in preeclamptic pregnancy to those observed in normal pregnancy [52]—though this contrasts with the findings of Stallmach [53]. It is proposed that these apparent contradictions between studies using immunological detection of cytokines and bioactivity studies may reflect changes in cytokine inhibitory binding proteins during preeclamptic pregnancy [54].

Numerous reports indicate that the plasma of preeclamptic patients contains elevated levels of IL-6, a multifunctional cytokine that regulates, among other things, the acute phase reaction and modulates both pro- and anti-inflammatory events, and may play roles in the pathogenesis of preeclampsia by serving as a source of a key circulating factor that promotes systemic maternal endothelial cell dysfunction [55]. Additionally, while many of the functions of IL-6 have not been explained yet, it is assumed that IL-6 is a good biomarker for adverse pregnancies [56]. In this study it was found that all tested compounds, i.e., methyldopa and all flavonoids, increased mRNA IL-6 expression, but every combined administration of a flavonoid together with methyldopa showed a strong contrary inhibitory effect when compared to the value for methyldopa in JEG-3 cells. At the protein level, no significant changes were found for either individual substances or their combinations with methyldopa. In HUVEC cells we observed similar effects at the mRNA level as for JEG-3 cells. However, at the protein level all compounds were found to increase IL-6, but after their combined administration, there were inhibitory effects for all flavonoids compared to the value for methyldopa. Summarizing these results, it can be stated that even if methyldopa and individual flavonoids increased the activity of the formation of this cytokine, the inhibitory effect was generally observed after combined administrations of flavonoids with this drug, which may have a positive effect.

A number of growth factors/cytokines with angiogenic properties have been actively studied in the context of preeclampsia, including HIF-1α, PIGF, TGF-β, and VEGF.

The TGF-β superfamily includes inhibins, activins, bone morphogenic peptides, and growth and differentiation factors. It has been established that TGF-β is one of the cytokines with expression in macrophages and epithelial tissue [57], for example, in asthmatic epithelium [58], and moreover, it is associated with preeclampsia risk [54,59]. It is also well known that the increased TGF β-1 level may lead to preeclampsia [60,61]. In this study, the effect of administering all substances was similar and did not significantly affect mRNA TGF-β expression in relation to the control group, but combined treatment using flavonoids with methyldopa lowered the expression in relation to the methyldopa group in JEG-3 cells. Similarly, at the protein level the administration of flavonoids together with methyldopa showed a general inhibitory effect on the action of the flavonoids alone, and the strongest inhibitory effect was noted for methyldopa + quercetin when compared to methyldopa alone. In HUVEC cells, especially after treatments of different flavonoids combined with methyldopa, at both the mRNA and protein levels, a general inhibitory effect in relation to the action of methyldopa alone was also found. Summarizing the above, the combined treatments of flavonoids and methyldopa lowered both TGF-β mRNA and its protein level in both types of cells, which is a positive sign for their possible use in preeclampsia.

The VEGF family, in particular, has been of great interest, due to its known association with hypertension and nephropathy, and its role as a biomarker of endothelial dysfunction, platelet activation and tissue hypoxia [62]. These angiogenic factors are also potent mediators of the inflammatory response, and they augment inflammatory symptoms in patients with preeclampsia [45]. VEGF as a proangiogenic factor needs consideration as a biomarker associated with endothelial cell damage in pregnancy with severe preeclampsia [63]. However, in patients, the VEGF level in the PIH group was significantly lower than in the pregnancy group at advanced pregnancy, and the VEGF level significantly and gradually decreased with PIH aggravation; therefore, its role is not simple [64]. In this study we found that all the tested compounds, i.e., methyldopa and all flavonoids, act in a similar way, significantly increasing mRNA VEGF expression in JEG-3 cells, but every combination of flavonoids significantly lowered the expression in relation to the methyldopa group, showing an inhibitory effect, which was most apparent for the combinations methyldopa + quercetin and methyldopa + chrysin. At the protein level, generally the flavonoids and methyldopa decreased the level of VEGF, and the administration of methyldopa with quercetin also showed a strong inhibitory effect in relation to the action of methyldopa alone. Moreover, similar results were shown in HUVEC cells, and the strongest inhibitory effect in relation to methyldopa was obtained for the combination of methyldopa and quercetin at both the mRNA and protein levels. Since methyldopa decreased the VEGF level in severe preeclampsia patients by 10% at the dose of 250 mg and by 57% at the dose of 500 mg [63], it can be assumed that administering methyldopa together with flavonoids, especially quercetin, will allow lower doses of the drug to be used, which may be beneficial in reducing the risk of side effects from methyldopa.

PIGF is a VEGF related molecule that is expressed at high levels by trophoblast cells in the placenta [62] and it is known to play an important role in the pathophysiology of preeclampsia [65]. It is known that the rise in plasma PIGF levels observed in normal pregnancies is significantly attenuated in pregnancies complicated by preeclampsia [66]. In our study, methyldopa and all flavonoids acted at a similar level by increasing mRNA PIGF expression, but only methyldopa and apigenin showed statistically significant increases in JEG-3 cells. Their combined treatment did not produce significant differences in relation to the values for methyldopa, but at the protein level the administration of methyldopa with all flavonoids increased the level of PIGF in relation to the effect of methyldopa. In HUVEC, methyldopa and all flavonoids increased mRNA expression similarly, and the strongest effect was noted for methyldopa and scutellarin. On the other hand, every combination of flavonoids significantly lowered the expression in relation to the methyldopa group, and the strongest inhibitory effect was found for the combination methyldopa + quercetin. At the protein level, similar effects were found. It could be stated that, while the effect of individual compounds is positive and increases the expression and level of this factor, the combined administration of methyldopa with flavonoids is rather unfavorable. Nevertheless, the effect of methyldopa on PIGF is not entirely clear, since there are even some data suggesting that methyldopa may have a specific effect on placental and/or endothelial cell function in preeclampsia patients, altering angiogenic proteins; however, the drug did not change the level of PIGF in women with preeclampsia [67].

HIF-1α is a transcription factor that is regulated by hypoxia and mediates the effects of hypoxia on gene expression [68]. It is expressed in the placenta in a gestational-age dependent fashion, with levels being higher in the first trimester and declining as oxygen levels increase later in pregnancy [54]. Accumulation of HIF-1α is commonly an acute and beneficial response to hypoxia; when chronically elevated, this protein is associated with multiple disease conditions, including preeclampsia [69]. There are some data indicating that women with preeclampsia are characterized by persistently elevated placental HIF-1α levels [70,71]. In this study we found that all the compounds, i.e., methyldopa and all flavonoids, increased mRNA HIF1-α expression, but only for methyldopa was the effect statistically significant in JEG-3 cells; however, the combined administrations of flavonoids with methyldopa lowered the expression in relation to the group from methyldopa, and the strongest inhibitory effects were observed for the combinations of methyldopa + baicalein and methyldopa + chrysin. Similar effects were observed at the protein level, and the strongest inhibitory effect was found for the combination methyldopa + quercetin. In HUVEC, methyldopa and all flavonoids increased HIF-1αmRNA and protein, but as for JEG-3 cells, their combined treatments with methyldopa produced inhibitory effects, leading to values similar to the control. Summarizing the above, it can be stated that even if methyldopa and individual flavonoids increased the activity of the formation of this factor, an inhibitory effect was generally observed after a combined administration of a flavonoid with this drug, which may have a positive effect.

In conclusion, it was found that every combination of a flavonoid and methyldopa in these cells induced a down-regulating effect onevery factor tested, except PIGF, especially at the mRNA level. Since it is known that hypertension generally raises TNF-α, IL-1β, IL-6, HIF-1α, TGF-β, and VEGF mRNA and/or protein levels, the results obtained in the study may provide a positive prognostic factor for such activity in vivo.

## 4. Materials and Methods

### 4.1. Materials

Methyldopa, apigenin, baicalein, chrysin, quercetin, and scutellarin were provided by Sigma-Aldrich (Poznan, Poland). Methyldopa was used as a reference substance.

### 4.2. Cell Lines and Culture Conditions

The primary human umbilical vein endothelial cell line (HUVEC, ATCC CRL1730) and trophoblast-derived human choriocarcinoma cell line (JEG-3, ATCCHTB-36) were obtained from American Type Culture Collection (ATCC). The cell lines were cultured in an incubator at 37 °C with a 5% CO_2_ atmosphere with 95% humidity. JEG-3 cells were cultured in Dulbecco’s modified eagle medium (DMEM) (Sigma-Aldrich, Poznan, Poland). The medium was enriched with 10% fetal bovine serum (FBS) (Sigma-Aldrich Poznan, Poland) and 0.1% penicillin (100 U/mL)/streptomycin (100 µg/mL) (Sigma-Aldrich, Poznan, Poland). HUVEC were cultured using Vascular Cell Basal Medium and Endothelial Cell Growth KitBBE (ATCC PCS100040).

All flavonoids and methyldopa were separately applied to HUVEC and JEG-3 cells in chosen concentrations (20, 40, and 100 µmol). The concentrations of compounds were selected on the basis of our preliminary experience and similar work [72]. The compounds were dissolved in dimethyl sulfoxide (DMSO); the content of DMSO was kept below 0.1%, as this concentration was found to be nontoxic to the cell lines.

After selecting the concentration least influencing cell viability, i.e., 40 µmol, in the second part of the experiment all compounds were administered either alone or in combination with methyldopa.

Each cell line (HUVEC, JEG-3) was treated with the tested compounds for 48 h at 37 °C, with a 5% CO_2_ atmosphere. The cell growth was analyzed by counting viable cells in the presence of trypan blue (Sigma-Aldrich, Poznan, Poland) with a Bucker hemocytometer (Sigma-Aldrich Poznan, Poland). To determine the antiproliferative activity for substances studied, we performed the MTT assay adding 10 µL of a MTT (3-(4,5-dimethylthiazol-2-yl)-2,5-diphenyltetrazolium bromide solution (concentration 5 mg/mL) (Sigma-Aldrich, Poznan, Poland) and then incubating for 4 h at 37 °C. The viable cells were visualized by the development of purple color due to the formation of formazan crystals which were dissolved with 100 µL of isopropyl alcohol at 0.05 N HCl. Next, the absorbance was measured at 570 nm on a microplate reader (Infinite 200, TECAN, Männedorf, Switzerland) using the wavelength of 655 nm as a reference [72].

### 4.3. Expression Analysis

The isolation of total cellular RNA was performed according to the manufacturer’s protocol of TriPure Isolation Reagent (Roche, Mannheim, Germany). The synthesis of complementary DNA was performed using the Transcriptor First-Strand Synthesis System (Roche, Mannheim, Germany) according to the manufacturer’s protocol. The obtained transcripts were used directly for the real-time PCR (RT-PCR) or stored at −20 °C.

The mRNA levels of studied genes (PIGF, VEGF, TNF-α, HIF-1α, TGF-β, IL-1β, and IL-6) were analyzed by real-time quantitative PCR using a LightCycler96 Instrument (Roche, Mannheim, Germany) and a LightCycler480 Probes Master kit (Roche, Mannheim, Germany). GAPDH was used as a housekeeping gene for normalization. The PCR program was initiated with an activation at 95 °C for 10 min. Each PCR cycle comprised a denaturation step at 95 °C, an annealing step at a specific temperature, and an extension step at 72 °C. The sequences of the primers were designed using the Oligo 4.0 program (National Biosciences, Colorado Springs, CO, USA,). All oligonucleotide sequences were synthesized by Sigma-Aldrich (Poznan, Poland) and are summarized in Table 5. The increase in fluorescence of PCR products was monitored and measured and the data were analyzed with the LightCycler96 software.

### 4.4. ELISA

After 48 h, cell culture supernatants were collected and stored at −30 °C. TGF-β secreted into the medium was detected by the TGF-β-1 Human ELISA Kit (sensitivity: 8.6 pg/mL; Life Technologies, Inc., Carlsbad, CA, USA). The HIF1α Human ELISA Kit (sensitivity: 30 pg/mL; Life Technologies Inc., Carlsbad, CA, USA), Human Placental Growth Factor ELISA Kit (sensitivity: 2 pg/mL; Sigma Aldrich, Poznan, Poland), Human IL-1β ELISA Kit (sensitivity: 0.5 pg/mL; Sigma Aldrich, Poznan, Poland), IL-6 Human ELISA Kit (sensitivity < 1 pg/mL; Life Technologies Inc., Carlsbad, CA, USA), Human VEGF ELISA Kit (sensitivity: 10 pg/mL; Sigma Aldrich, Poznan, Poland), and TNF-α Human ELISA Kit (sensitivity: 0.13 pg/mL; Life Technologies, Inc., Carlsbad, CA, USA) were employed to evaluate the secretions in the cell culture supernatant of these factors according to the manufacturers’ protocols (instructions): 100 µL of each standard and the same of each sample were added to the appropriate well. Wells were covered and incubated at room temperature. The solution was removed and washed several times with PBS. Then, 100 µL of antibody was added to each well. Wells were covered and incubated at room temperature with gentle shaking. Next the wells were washed as described above, and 100 µL of prepared streptavidin solution was added to each well. Wells were covered and incubated at room temperature with gentle shaking. Next the wells were washed again, and substrate reagent was added to each well and then the wells were covered and incubated in the dark at room temperature. The reaction was blocked and the absorbance was measured on a microplate reader (Infinite 200, TECAN). The concentrations of PIGF, VEGF, TNF-α, HIF-1α, TGF-β, IL-1β, and IL-6 were determined by interpolation of the standard curve using linear regression analysis.

## 5. Statistical Analysis

All values were expressed as mean ± SD. The statistical comparison of results was carried out using analysis of variance (ANOVA) followed by the Newman–Keuls test as a post-hoc test for detailed data analysis. The values of *p* < 0.05 were considered to indicate a statistically significant difference.

## Figures and Tables

**Table 1:** Influences of methyldopa and its combinations with individual flavonoids on the mRNA expression of selected factors related to inflammatory processes (TNF-α, IL-1β, and IL-6) and cardiovascular diseases (TGF-β, VEGF, PIGF, and HIF-1α) in JEG-3 cells.

Group	TNF-α	TGF-β	VEGF	PIGF	HIF-1α	IL-1β	IL-6
control	1.88 ± 0.04	1.4 ± 0.02	1.20 ± 0.05	1.20 ± 0.09	1.16 ± 0.06	1.83 ± 0.03	1.75 ± 0.11
methyldopa	1.53 ± 0.21 *	1.50 ± 0.13	1.65 ± 0.09 *	1.90 ± 0.21 *	1.29 ± 0.03 *	1.60 ± 0.13 *	1.92 ± 0.16
apigenin	1.97 ± 0.20	1.40 ± 0.18	1.54 ± 0.02 *^,^#	1.55 ± 0.05^*^	1.23 ± 0.04	1.55 ± 0.12 *	2.10 ± 0.17 *
Methyldopa + apigenin	0.90 ± 0.05 *^,^#	0.88 ± 0.03 *,#	0.86 ± 0.02 *^,^#	1.17 ± 0.01 *,#	0.95 ± 0.03 *^,^#	1.05 ± 0.04 *^,^#	1.11 ± 0.02 *^,^#
baicalein	1.53 ± 0.10 *	1.41 ± 0.13	1.40 ± 0.08	1.24 ± 0.12	1.22 ± 0.02	1.64 ± 0.07	2.06 ± 0.11 *
Methyldopa + baicalein	0.90 ± 0.04 *^,^#	0.75 ± 0.06 *^,^#	0.84 ± 0.05 *^,^#	1.05 ± 0.01 *^,^#	0.84 ± 0.02 *^,^#	1.00 ± 0.03 *^,^#	1.07 ± 0.03 *^,^#
chrysin	1.40 ± 0.13 *	1.47 ± 0.13	1.47 ± 0.16 *	1.48 ± 0.16	1.26 ± 0.02	1.50 ± 0.04 *	1.91 ± 0.01
methyldopa+ chrysin	0.79 ± 0.02 *^,^#	0.64 ± 0.01 *^,^#	0.72 ± 0.02 *^,^#	0.90 ± 0.01 *^,^#	0.82 ± 0.09 *^,^#	0.89 ± 0.03 *^,^#	1.09 ± 0.03 *^,^#
quercetin	1.56 ± 0.03 *	1.35 ± 0.04	1.38 ± 0.14	1.31 ± 0.14	1.25 ± 0.01	1.67 ± 0.09	1.86 ± 0.02
Methyldopa + quercetin	0.90 ± 0.05 *^,^#	0.88 ± 0.07 *^,^#	0.89 ± 0.06 *^,^#	1.16 ± 0.05 *^,^#	0.94 ± 0.02 *^,^#	1.09 ± 0.04 *^,^#	1.06 ± 0.02 *^,^#
scutellarin	1.56 ± 0.04 *	1.60 ± 0.05	1.58 ± 0.05 *	1.52 ± 0.07 *	1.26 ± 0.01	1.76 ± 0.05	2.30 ± 0.04 *
Methyldopa + scutellarin	0.78 ± 0.04 *^,^#	0.73 ± 0.03 *^,^#	0.82 ± 0.06 *^,^#	1.03 ± 0.01 *^,^#	0.93 ± 0.03 *^,^#	0.92 ± 0.04 *^,^#	1.06 ± 0.03 *^,^#

Values are presented as ratios against mRNA GAPDH expression. Mean ± SD. *—vs. control, *p* < 0.05.; #—vs. methyldopa, *p* < 0.05.

**Table 2:** Influences of methyldopa and its combinations with individual flavonoids, at the protein level, on selected factors related to inflammatory processes (TNF-α, IL-1β, and IL-6) and cardiovascular diseases (TGF-β, VEGF, PIGF, and HIF-1α) in JEG-3 cells.

Table. *Cont.*	TNF-α	TGF-β	VEGF	PIGF	HIF-1α	IL-1β	IL-6
	[ng/mL]						
control	0.041 ± 0.002	0.128 ± 0.002	0.174 ± 0.004	3.274 ± 0.055	0.011 ± 0.003	0.008 ± 0.001	0.035 ± 0.006
methyldopa	0.038 ± 0.003	0.095 ± 0.003 *	0.061 ± 0.002 *	3.006 ± 0.028 *	0.052 ± 0.004 *	0.010 ± 0.001	0.035 ± 0.002
apigenin	0.032 ± 0.002 *	0.118 ± 0.001 *	0.203 ± 0.004 *	3.340 ± 0.126	0.011 ± 0.002	0.007 ± 0.001	0.043 ± 0.004
Methyldpa + apigenin	0.029 ± 0.001 *^,^#	0.091 ± 0.001	0.115 ± 0.002 *^,^#	3.290 ± 0.001 #	0.025 ± 0.002 #	0.008 ± 0.001	0.046 ± 0.001
baicalein	0.039 ± 0.002	0.113 ± 0.003 *	0.138 ± 0.002 *	3.507 ± 0.031	0.020 ± 0.003	0.009 ± 0.002	0.051 ± 0.008 *
Methyldopa + baicalein	0.030 ± 0.001 *^,^#	0.087 ± 0.001 *^,^#	0.122 ± 0.002 *^,^#	3.405 ± 0.001 #	0.017 ± 0.002 #	0.019 ± 0.001 *^,^#	0.039 ± 0.001
chrysin	0.035 ± 0.002 *	0.145 ± 0.004 *	0.194 ± 0.004 *	3.325 ± 0.171	0.022 ± 0.003	0.009 ± 0.002	0.033 ± 0.004
methyldopa+ chrysin	0.033 ± 0.001 *	0.107 ± 0.002 *^,^#	0.144 ± 0.001 *^,^#	3.341 ± 0.001 #	0.015 ± 0.001 #	0.014 ± 0.001 *	0.043 ± 0.002
quercetin	0.027 ± 0.002 *	0.081 ± 0.002 *	0.131 ± 0.004 *	3.374 ± 0.038	0.019 ± 0.003	0.010 ± 0.001	0.031 ± 0.004
Methyldopa + quercetin	0.035 ± 0.001 *	0.080 ± 0.001 *^,^#	0.048 ± 0.001 *^,^#	2.798 ± 0.001 *	0.012 ± 0.001 #	0.013 ± 0.001	0.041 ± 0.002
scutellarin	0.038 ± 0.002	0.141 ± 0.002 *	0.152 ± 0.002 *	3.284 ± 0.160	0.020 ± 0.004	0.011 ± 0.003	0.041 ± 0.005
Methyldopa + scutellarin	0.028 ± 0.001 *^,^#	0.110 ± 0.002 *^,^#	0.091 ± 0.001 *^,^#	3.357 ± 0.001 #	0.027 ± 0.001 *^,^#	0.014 ± 0.001 *	0.040 ± 0.001

Mean ± SD. *—vs. control, *p* < 0.05. #—vs. methyldopa, *p* < 0.05.

**Table 3:** Influences of methyldopa and its combinations with individual flavonoids on the mRNA expression of selected factors related to inflammatory processes (TNF-α, IL-1β, and IL-6) and cardiovascular diseases (TGF-β, VEGF, PIGF, and HIF-1α) in HUVEC.

Group	TNF-α	TGF-β	VEGF	PIGF	HIF-1α	IL-1β	IL-6
control	1.17 ± 0.08	1.12 ± 0.06	1.12 ± 0.06	1.14 ± 0.06	1.12 ± 0.03	1.01 ± 0.03	1.26 ± 0.06
methyldopa	1.51 ± 0.27 *	1.54 ± 0.10 *	1.43 ± 0.01 *	1.85 ± 0.08 *	1.35 ± 0.05 *	1.42 ± 0.05 *	1.96 ± 0.04 *
apigenin	2.27 ± 0.12 *	1.50 ± 0.18 *	1.66 ± 0.16 *	1.59 ± 0.24 *	1.90 ± 0.06 *	1.75 ± 0.05 *	1.55 ± 0.04 *
Methyldopa + apigenin	1.37 ± 0.03	1.27 ± 0.05 #	1.36 ± 0.18	1.43 ± 0.09 *^,^#	1.16 ± 0.02 #	1.60 ± 0.10 *	1.15 ± 0.07 #
baicalein	1.64 ± 0.04 *	1.48 ± 0.02 *	1.44 ± 0.03 *	1.56 ± 0.11 *	1.60 ± 0.05 *	1.57 ± 0.07 *	1.44 ± 0.03 *
Methyldopa + baicalein	1.40 ± 0.09	1.21 ± 0.02 #	1.34 ± 0.14	1.43 ± 0.02 *^,^#	1.15 ± 0.03 #	1.59 ± 0.01 *	1.10 ± 0.04 *^,^#
chrysin	1.52 ± 0.01 *	1.57 ± 0.07 *	1.40 ± 0.01 *	1.50 ± 0.07 *	1.50 ± 0.06 *	1.36 ± 0.07 *	1.41 ± 0.01 *
Methyldopa + chrysin	1.16 ± 0.05 #	1.16 ± 0.08 #	1.36 ± 0.10	1.36 ± 0.03 *^,^#	1.12 ± 0.01 #	1.70 ± 0.05 *^,^#	1.08 ± 0.01 *^,^#
quercetin	1.63 ± 0.03 *	1.41 ± 0.04 *	1.43 ± 0.08 *	1.40 ± 0.05	1.52 ± 0.04 *	1.32 ± 0.04 *	1.55 ± 0.04 *
Methyldopa + quercetin	1.15 ± 0.15 #	1.17 ± 0.04 #	1.30 ± 0.04	1.30 ± 0.07 #	1.02 ± 0.01 #	1.52 ± 0.06 *	1.02 ± 0.01 *^,^#
scutellarin	1.63 ± 0.04 *	1.70 ± 0.10 *	1.69 ± 0.04 *	1.69 ± 0.10 *	1.66 ± 0.06 *	1.44 ± 0.05 *	1.40 ± 0.04 *
Methyldopa + scutellarin	1.14 ± 0.03 #	1.23 ± 0.14 #	1.30 ± 0.10	1.38 ± 0.07 #	1.14 ± 0.04 #	1.35 ± 0.14 *	1.12 ± 0.02 *^,^#

Values are presented as ratios against mRNA GAPDH expression. Mean ± SD. *—vs. control, *p* < 0.05. #—vs. methyldopa, *p* < 0.05.

**Table 4:** Influences of methyldopa and its combinations with individual flavonoids, at the protein level, on selected factors related to in Figure 1. and IL-6) and cardiovascular diseases (TGF-β, VEGF, PIGF, and HIF-1α) in HUVEC.

Group	TNF-α	TGF-β	VEGF	PIGF	HIF-1α	IL-1β	IL-6
	[ng/mL]						
control	0.046 ± 0.001	0.150 ± 0.001	0.162 ± 0.001	3.398 ± 0.002	0.017 ± 0.001	0.015 ± 0.001	0.050 ± 0.001
methyldopa	0.040 ± 0.001 *	0.117 ± 0.002 *	0.161 ± 0.001	3.393 ± 0.002	0.025 ± 0.003 *	0.011 ± 0.001	0.057 ± 0.001
apigenin	0.041 ± 0.001 *	0.144 ± 0.001	0.201 ± 0.001 *	3.499 ± 0.002 *	0.022 ± 0.001	0.018 ± 0.001	0.061 ± 0.002 *
Methyldopa + apigenin	0.031 ± 0.002 *^,^#	0.090 ± 0.002 *^,^#	0.116 ± 0.002 *^,^#	3.293 ± 0.003 *^,^#	0.020 ± 0.001	0.009 ± 0.001 *	0.042 ± 0.001 *^,^#
baicalein	0.038 ± 0.001 *	0.169 ± 0.001 *	0.155 ± 0.003	3.564 ± 0.002 *	0.025 ± 0.001 *	0.017 ± 0.002	0.069 ± 0.001 *
Methyldopa + baicalein	0.027 ± 0.001 *^,^#	0.080 ± 0.002 *^,^#	0.121 ± 0.002 *^,^#	3.410 ± 0.011	0.019 ± 0.001	0.017 ± 0.001 #	0.037 ± 0.004 *^,^#
chrysin	0.049 ± 0.001	0.152 ± 0.002	0.165 ± 0.001	3.505 ± 0.003^*^	0.030 ± 0.001^*^	0.018 ± 0.001	0.063 ± 0.001^*^
Methyldopa + chrysin	0.037 ± 0.002 *	0.110 ± 0.001 *^,^#	0.135 ± 0.002 *^,^#	3.309 ± 0.011 *^,^#	0.014 ± 0.001	0.014 ± 0.002	0.040 ± 0.001 *^,^#
quercetin	0.035 ± 0.002 *	0.096 ± 0.001 *	0.167 ± 0.001	3.204 ± 0.002 *	0.020 ± 0.001	0.014 ± 0.001	0.063 ± 0.002 *
Methyldopa + quercetin	0.032 ± 0.001 *^,^#	0.081 ± 0.002 *^,^#	0.046 ± 0.005 *^,^#	2.805 ± 0.014 *^,^#	0.014 ± 0.004	0.013 ± 0.003	0.039 ± 0.006 *^,^#
scutellarin	0.044 ± 0.001	0.154 ± 0.002	0.161 ± 0.001	3.491 ± 0.001 *	0.024 ± 0.001 *	0.020 ± 0.001 *	0.071 ± 0.002 *
Methyldopa + scutellarin	0.030 ± 0.001 *^,^#	0.130 ± 0.001 *^,^#	0.093 ± 0.004 *^,^#	3.344 ± 0.002 *^,^#	0.030 ± 0.001 *^,^#	0.014 ± 0.002	0.040 ± 0.001 *^,^#

Mean ± SD. *—vs. control, *p* < 0.05. #—vs. methyldopa, *p* < 0.05.

**Table 5:** Sequences of primers used for the RT-PCR analysis.

Gene	Primer Sequence Forward (5ʹ→3ʹ)	Primer Sequence Reverse (5ʹ→3ʹ)	bp
TNF-α	TGC TTG TTC CTC AGC CTC TT	TGA GGT ACA GGC CCT CTG AT	218
IL-1β	CGA TGC ACC TGT ACG ATC AC	TCT TTC AAC ACG CAG GAC AG	226
HIF-1α	TTG CCT TTC CTT CTC TTC TCC	CAA TCC AAG GTT GCC AAG TT	164
VEGF	CCT TGC TGC TCT ACC TCC AC	ATC CAC CCC AAA ACT TTT CC	236
TGF-β	ACA TTG ACT TCC GCA AGG AC	CCG GGT TAT GCT GGT TGT A	150
PIGF	GTT CAG CCC ATC CTG TGT CT	AGC AGG GAA ACA GTT GGC TA	244
IL-6	TGC GTC CGT AGT TTC CTT CT	GGA ATC TTC TCC TGG GG GTA	211
GAPDH	GAT GGT GAA GGT CGG TGT G	ATG AAG GGG TCG TTG ATG G	108

GAPDH—glyceraldehyde 3-phosphate dehydrogenase.

## Data Availability

The data presented in this study are available in the article.

## Sample Availability

Samples of the compounds are available from the authors.

## Abbreviations

ACE	angiotensin-converting enzyme
AT1	II receptor
DMSO	dimethyl sulfoxide
eNOS	endothelial nitric oxide synthase
GAPDH	glyceraldehyde 3-phosphate dehydrogenase
HIF1α	hypoxia-inducible factor 1α
HUVEC	human umbilical vein endothelial cells
IL1β	interleukin-1β
IL6	interleukin 6
JEG-3 cells	trophoblast-derived human choriocarcinoma cells
mRNA	messenger RNA
MTT	3-(4,5-dimethylthiazol-2-yl)-2,5-diphenyltetrazolium bromide
PCR	polymerase chain reaction
PIH	pregnancy-induced hypertension
PIGF	placental growth factor
qPCR	quantitative polymerase chain reaction
qRT-PCR	quantitative reverse transcription polymerase chain reaction
TGF-β	transforming growth factor β
TNF-α	tumor necrosis factor α
VEGF	vascular endothelial growth factor
ACE	angiotensin-converting enzyme
AT1	II receptor
DMSO	dimethyl sulfoxide
eNOS	endothelial nitric oxide synthase
